# PME: pruning-based multi-size embedding for recommender systems

**DOI:** 10.3389/fdata.2023.1195742

**Published:** 2023-06-15

**Authors:** Zirui Liu, Qingquan Song, Li Li, Soo-Hyun Choi, Rui Chen, Xia Hu

**Affiliations:** ^1^Computer Science Department, Rice University, Houston, TX, United States; ^2^Linkedin, Sunnyvale, CA, United States; ^3^Samsung Electronics America, Mountain View, CA, United States

**Keywords:** neural network, recommender system, embedding compression, pruning, scalability

## Abstract

Embedding is widely used in recommendation models to learn feature representations. However, the traditional embedding technique that assigns a fixed size to all categorical features may be suboptimal due to the following reasons. In recommendation domain, the majority of categorical features' embeddings can be trained with less capacity without impacting model performance, thereby storing embeddings with equal length may incur unnecessary memory usage. Existing work that tries to allocate customized sizes for each feature usually either simply scales the embedding size with feature's popularity or formulates this size allocation problem as an architecture selection problem. Unfortunately, most of these methods either have large performance drop or incur significant extra time cost for searching proper embedding sizes. In this article, instead of formulating the size allocation problem as an architecture selection problem, we approach the problem from a pruning perspective and propose **P**runing-based **M**ulti-size **E**mbedding (PME) framework. During the search phase, we prune the dimensions that have the least impact on model performance in the embedding to reduce its capacity. Then, we show that the customized size of each token can be obtained by transferring the capacity of its pruned embedding with significant less search cost. Experimental results validate that PME can efficiently find proper sizes and hence achieve strong performance while significantly reducing the number of parameters in the embedding layer.

## 1. Introduction

Embedding feature information into vector representations is crucial for the success of deep learning based recommendation models (Zhang et al., 2019). In practice, the input features to recommender systems are often categorical, such as userID, itemID, and the category of items. For deep learning based recommendation models, these categorical features are mapped to low-dimensional learnable vectors (i.e., embeddings). Then, the learned vectors are fed into the rest of the model to learn the interaction between features. The number of layers in the rest of the recommendation model is typically small (usually less than 10) and independent of the number of categorical features (Cheng et al., 2016; Guo et al., 2017; Lian et al., 2018). In contrast, the dimension of the embedding matrix grows linearly with the number of categorical features, which can easily be at the scale of millions (Park et al., 2018). As a result, the weight matrix of the embedding layer is often responsible for the major memory consumption of a deep learning based recommendation models. For example, the embedding layer of Facebook recommender system contains billions of parameters. Consequently, the embedding layer occupies more than 99.9% memory of the whole model, which can consume hundreds of gigabytes or even terabytes (Park et al., 2018; Ginart et al., 2021). Without compressing the embedding layers, the excessive memory usage of recommendation models is a major obstacle for serving them on-device, where the memory is limited.

Traditional embedding compression methods usually put efforts on compacting the embedding matrix (Markovsky and Usevich, 2012; Wang et al., 2017): Low-rank based methods assume the weight matrix has reduced rank that can be decomposed into several smaller matrices (Markovsky and Usevich, 2012). Hashing based methods reduce the number of embedding vectors in the matrix by mapping similar items into a same bucket (Wang et al., 2017). All these methods follow the framework of the standard embedding technique that learns embeddings with equal length for each token.^1^ However, recent advances demonstrate that assigning a fixed embedding size to all tokens may be suboptimal due to the following reasons (Joglekar et al., 2020; Zhao et al., 2020a,b; Ginart et al., 2021). In the recommendation domain, usually a few head tokens dominate the data, while the majority of tokens (i.e., long-tail tokens) are rarely observed (Park and Tuzhilin, 2008). Since the token's popularity and the importance of its representation to model performance is correlated (Joglekar et al., 2020; Zhao et al., 2020a; Ginart et al., 2021). Thus, when using a fixed embedding size, it may either lose the information of head tokens or waste parameters on long-tail tokens (Kang et al., 2020; Zhao et al., 2020b). We usually choose a large enough embedding size to ensure model performance, which incurs unnecessary memory usage for storing long-tail token's embedding.

To overcome the mentioned drawback of embedding with equal length, several recent work proposes to allocate more capacity (i.e., larger embedding size) to important tokens, and less capacity to unimportant ones (Joglekar et al., 2020; Kang et al., 2020; Zhao et al., 2020a,b; Ginart et al., 2021). These work can be roughly divided into two categories. Some work proposes to explicitly scale token's embedding size with its frequency according to heuristic rules designed by human experts (Kang et al., 2020; Ginart et al., 2021). However, such allocation strategy may be suboptimal since the importance of a token is not purely decided by its popularity. Inspired by neural architecture search (NAS), another line of research formulates the embedding size allocation problem as an architecture selection problem, which selects the embedding size for each token from several predefined options (Joglekar et al., 2020; Zhao et al., 2020a,b). Due to the extremely large search space, the search process incurs a significant computational cost. Although the number of parameters in the embedding layer is significantly reduced, these methods still either have large performance drop or introduce significant extra time cost for searching embedding sizes.

In this article, we approach the embedding size allocation problem from a pruning perspective. Our work is motivated by the observation that the majority of token's embeddings can be trained with less capacity without impacting model performance (Joglekar et al., 2020). Therefore, during the search phase, instead of selecting from a set of candidate embedding sizes, we prune the dimensions that have the least impact on model performance in token's embeddings to reduce its capacity. Then, we build a multi-size embedding table for training without sacrificing model performance, where the customized size of each token is obtained by transferring the capacity of its pruned embedding. Moreover, we show that the unimportant parameters in the embedding layer can be identified and pruned at initialization, and this significantly reduces the time cost of searching the customized sizes. Consequently, our framework can reduce the memory occupied by the embedding layer during both the training and inference phases without sacrificing model performance. Our contributions are summarized as follows:

- We rigorously show that the embedding size allocation problem can be converted to a pruning problem. Based on this reformulation, we propose a pruning-based multi-size embedding (PMB) framework to search the customized embedding size for each token.
- In our framework, during the search process, the embedding layer is pruned without training it. Thus, the time cost of the search process is significantly reduced. Once pruned, we build the multi-size embedding table for training by transferring the capacity of token's pruned embedding. Our framework can reduce the memory occupied by the embedding layer during both the training and inference phases.
- We show that our framework can match or improve the performance of several recommendation models using significantly less parameters. e.g., for Autoint+ (Song et al., 2019), we show that PME could significantly improve the Logloss and AUC while using 40 × fewer parameters for click-through rate prediction task on the Criteo dataset.

## 2. Preliminary and problem statement

### 2.1. Notations

We denote matrices with uppercase bold letters (e.g., **V**), vectors with lowercase bold letters (e.g., **v**), and scalars with lowercase alphabets (e.g., *v*). We use **V**_*i*, :_ to represent the *i*^th^ row of **V**, and **V**_*i,j*_ to denote the entry at the *i*^th^ row and *j*^th^ column of **V**. We denote the standard *L*_0_ norm as ||·||_0_. The operation **V** = concat(**V**_1_, **V**_2_) represents row-wisely concatenating matrix **V**_1_ and **V**_2_ into a new matrix **V**. We use ℕ = {0, 1, 2, 3⋯ } to denote the set of all non-negative natural numbers. We use ⊙ to denote the Hadamard product.

### 2.2. Preliminary

Recommender systems involve a massive amount of categorical feature fields, such as userIDs, itemIDs, and the category of items. Let **x** = [**x**_1_; **x**_2_; ⋯ ;**x**_*M*_] be an input instance with *M* feature fields, where **x**_*i*_ is the one-hot vector corresponding to the *i*^th^ field. Suppose the vocabulary size of the *i*^th^ field is *n*_*i*_, i.e., there are *n*_*i*_ unique tokens (i.e., categorical features) in the *i*^th^ field. For each token *x*_*i*_, it is mapped into a low-dimensional vector ${\text{v}}_{i}\in {\mathbb{R}}^{d}$ by **v**_*i*_ = **V**_*i*_**x**_*i*_, where ${\text{V}}_{i}\in {\mathbb{R}}^{n_{i}\times d}$ is the embedding matrix of the *i*^th^ field and *d* is the embedding size. For convenience of notations, let **V** = concat(**V**_1_, ⋯ , **V**_*M*_) be the embedding matrix consisting of all tokens' embeddings. Consider a deep learning based recommender system ϕ parameterized by **V** and Θ, where Θ denotes all other model's parameters excluding those in **V**. We denote the prediction corresponding to **x** as *ŷ* = ϕ(**x**|**V**, Θ). We aimed to minimize the loss L(**V**, Θ; D) 𝔼 *E*_(**x**, *y*)~D_ℓ(ϕ(**x**|**V**, Θ), *y*) over a dataset D = {(**x**, *y*)}, where ℓ is the loss function such as Logloss.

### 2.3. Multi-size embedding

The multi-size embedding framework allows each token in the vocabulary to have embeddings of different sizes (Joglekar et al., 2020; Ginart et al., 2021). By allocating an appropriate size for each token, the multi-size embedding framework can significantly reduce the total number of parameters in the embedding layer while maintaining the quality of learned representations (Joglekar et al., 2020). Although the multi-size embedding has the mentioned advantages over the standard single-size embedding, applying it requires solving the following problem: Suppose there are *n* tokens in the vocabulary. If the total number of parameters in the multi-size embedding table is limited to no more than a predefined budget *k*, how to search for the optimal size *d*_*i*_ of token *i* under the budget constraint, such that the loss could be minimized as much as possible with the learned *d*_*i*_-dimensional embedding vector ${\hat{\text{v}}}_{i}$? We formally define this embedding size allocation problem in Problem 1.

Problem 1 (Embedding size allocation problem). Given a maximum embedding size *d* and a predefined parameter budget *k*, let the ${\hat{\text{v}}}_{i}$ be a *d*_*i*_-dimensional embedding representing token *i*. For element-wise operations between embeddings to work, embeddings of different sizes are padded to equal length *d* with zeros following by a projection. Namely, the ${\hat{\text{v}}}_{i}\in {\mathbb{R}}^{d_{i}}$ will be padded with *e*_*i*_ trailing zeros such that *d*_*i*_+*e*_*i*_ = *d*, leading to a padded vector ${\hat{\text{v}}}_{i}^{\prime }\in {\mathbb{R}}^{d}$. We define **d** = [*d*_1_, ⋯ , *d*_*n*_]. Let $\hat{\text{V}}\in {\mathbb{R}}^{n\times d}$ be the single-size embedding matrix consisting of all projected *d*-dimensional embeddings, i.e., ${\hat{\text{V}}}_{i,:}={\text{P}}_{i}{\hat{\text{v}}}_{i}^{\prime }$, where ${\text{P}}_{i}\in {\mathbb{R}}^{d\times d}$ is a learnable projection matrix associated with token *i*. The goal of embedding size allocation problem aimed to solve the following optimization problem:


(1) $$\begin{array}{l} \underset{\text{d}}{\min}L\left({\hat{\text{V}}}^{*}\left(\text{d}\right),\Theta^{*}\left(\text{d}\right);D\right), \end{array}$$

(2) $$\begin{array}{ll} s.t.\; & {\hat{\text{V}}}^{*}\left(\text{d}\right),\Theta^{*}\left(\text{d}\right)=\underset{\hat{\text{V}},\Theta }{\mathrm{argmin}}L\left(\hat{\text{V}}\left(\text{d}\right),\Theta \left(\text{d}\right);D\right), \end{array}$$

(3) $$\begin{array}{l} \overset{n}{\underset{i=1}{\sum }}d_{i}\leq k, \end{array}$$

(4) $$\begin{array}{l} \forall i\in \left\{1,\cdots \;,n\right\},d_{i}\in \mathbb{N},d_{i}\leq d. \end{array}$$

**Figure 1:**
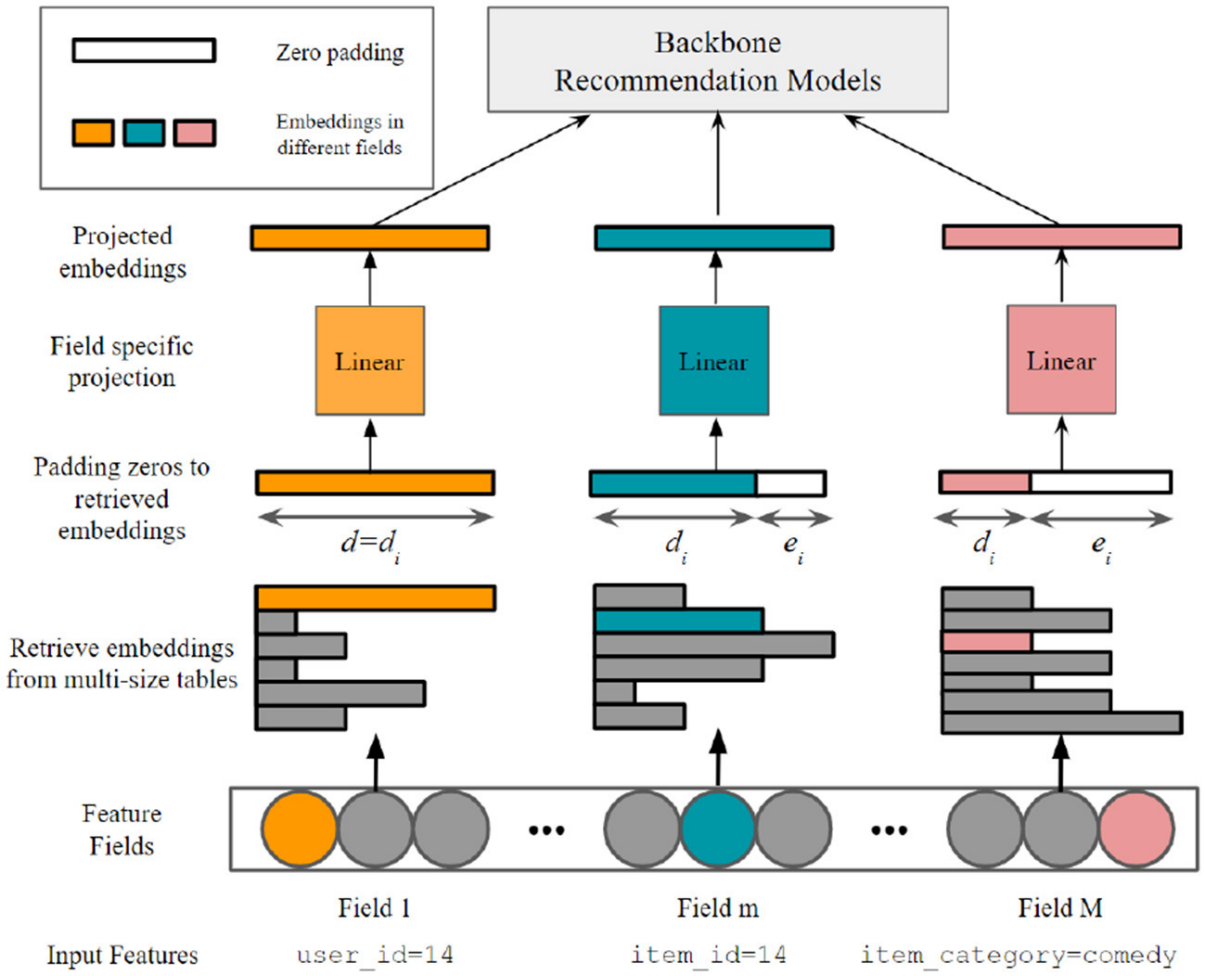
The multi-size embedding framework in our article. For element-wise operations to work (e.g., dot-product in factorization machines), the retrieved embeddings are padded to equal length with zeros following by a field-specific projection.

Figure 1 illustrates our multi-size embedding framework. The backbone recommendation models in Figure 1 refer to the rest of the model excluding the embedding layer. Although the projected embeddings have the same number of parameters as the uncompressed ones, we will only retrieve and project the embeddings for tokens in the current mini-batch data. As the mini-batch size restricts the number of retrieved embeddings, the memory usage from these additional parameters is negligible when considering the significant reduction in parameter numbers of the multi-size embedding table.

Following the studies by Zhao et al. (2020a) and Ginart et al. (2021), in our article, the projection matrix **P** in Problem 1 is shared between tokens in a same field to learn field-level structures. We note that such approach also has a nice algebraic explanation: the degree of freedom of the token *i*'s representation is limited by *d*_*i*_ since


(5) $$\begin{array}{ll} \text{P}{\hat{\text{v}}}_{i}^{\prime } & ={\left[\begin{array}{c} -{\text{p}}_{1}-\\ \cdots \\ -{\text{p}}_{d}- \end{array}\right]}_{d\times d}{\left[\begin{array}{c} v_{1};\cdots \;;v_{d_{i}};\underset{e_{i}}{\underset{︸}{0;\cdots \;;0}} \end{array}\right]}_{d\times 1}=\overset{d_{i}}{\underset{j=1}{\sum }}v_{j}{\text{p}}_{j}. \end{array}$$

In each field, for the token allocated with larger *d*_*i*_, the expressive ability of its embedding is stronger since it is represented using more basis from the row space of **P**. Thus, the multi-size embedding framework illustrated in Problem 1 can control the capacity of each token's representation by allocating different embedding sizes.

Solving Problem 1 poses a significant computational hurdle due to the following two reasons. First, in the recommendation domain, the vocabulary size can easily reach the million level (Covington et al., 2016). Second, since the size of embedding could only be integers, the combinatorial nature of this problem leads to an intractable optimization for a large search space. Finding the optimal embedding sizes for millions of tokens from a discrete search space requires a large amount of computational resources.

In the next section, we show that this combinatorial optimization problem can be converted to a pruning problem, which can be approximately solved with significantly less cost.

## 3. Methodology

**Figure 2:**
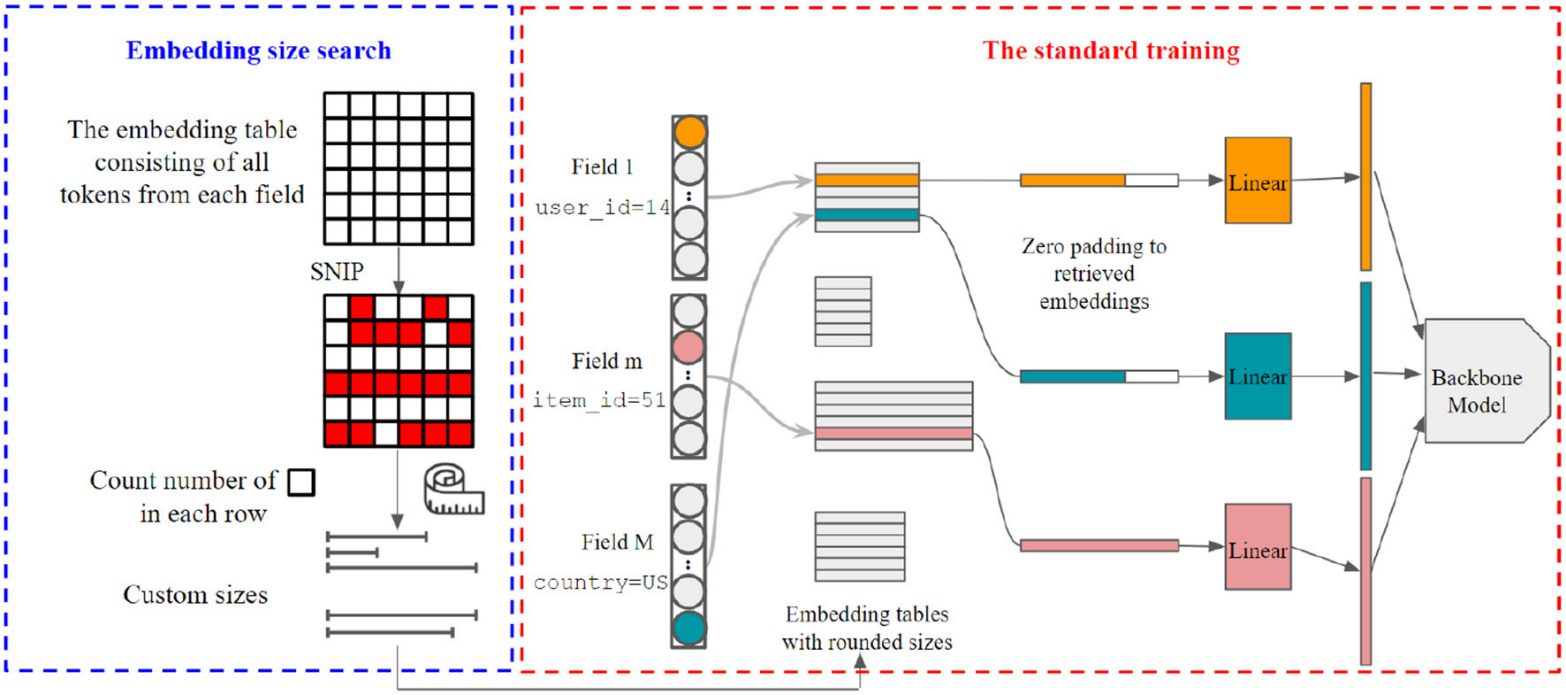
Overview of PME framework.

Figure 2 illustrates the overview of our proposed framework. We first search the customized embedding size for each token in a separate search process before training. The key intuition of our proposed method is the optimal capacity of a token that can be obtained by pruning unimportant dimensions in its embedding. In particular, given a standard single-size embedding layer, we prune the dimensions that have the least impact on model performance in token's embeddings to reduce its capacity. Then, the customized size of each token can be obtained by transferring the capacity of its pruned embedding (Section 3.1). We then derive our proposed pruning-based multi-size embedding framework, which prunes the embedding layer at initialization (Section 3.2). In this way, the time cost of the search process is significantly reduced.

In practice, a multi-size table is implemented as multiple two-dimensional embedding matrices, each with different sizes. Since the searched size could be any integer smaller than the maximal size *d*, we need to initialize at most *d* two-dimensional matrices, which incurs extra time cost to the retrieval process. To reduce the extra time cost of retrieving from multi-size table, we optimize the retrieval process based on group-wise operations (Section 3.3).

### 3.1. Size allocation as a pruning problem

**Figure 3:**
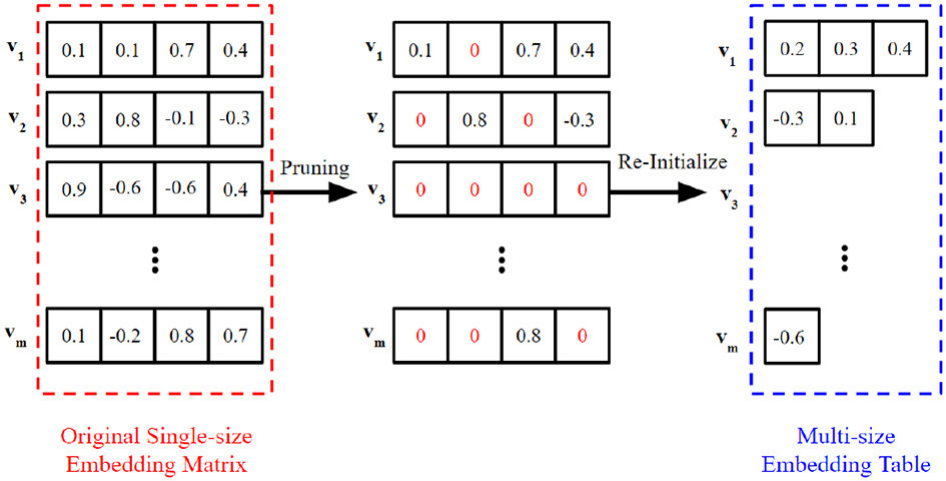
An example to illustrate the pruning-based multi-size embedding. After pruning, we build the multi-size embedding table for training, where the size of each token is set to the number of remaining parameters in its pruned embedding. We note that some tokens may be entirely cutoff from the vocabulary (such as **v**_3_, in this example), and they are mapped to unlearnable zero vectors.

The success of multi-size embedding framework suggests the embeddings of long-tail tokens can be trained with less capacity without impacting model performance (Joglekar et al., 2020; Ginart et al., 2021). This implies that there exists redundant parameters in the single-size embedding. It is intuitive to start pruning from the parameters that have the least impact on model performance, which is equivalent to reducing the embedding size. For example, as shown in Figure 3, the second value in embedding **v**_1_ is pruned out and set as zero, leading to a *d*_1_ = *d*−1 embedding size in effect. The actual size of the pruned embedding equals the number of remaining parameters.

Informally, by setting token *i*'s allocated size *d*_*i*_ to the number of remaining parameters, the capacity of its pruned embedding will be transferred to ${\hat{\text{v}}}_{i}$ in Problem 1. We formalize this statement by showing under mild assumptions, the optimal solution of Problem 1 can be constructed using the pruned embeddings 2. We first give the definition of redundant parameter identification problem.

Problem 2 (Redundant parameters identification problem). Given an overparameterized embedding matrix **V**∈ℝ^*n*×*d*^, the redundant parameter identification problem aims to solve the following constrained optimization problem:


(6) $$\begin{array}{l} \underset{\text{V},\Theta ,\text{C}}{\min}L\left(\text{V}⊙\text{C},\Theta ;D\right), \end{array}$$

(7) $$\begin{array}{l} s.t.\;\text{C}\in {\left\{0,1\right\}}^{n\times d},||\text{C}|{|}_{0}\leq k, \end{array}$$

where **C** is an auxiliary variable representing binary “gates” that denotes whether a parameter in *V* is present. *k* is the parameter budget referring to the number of non-zero entries in **V**, i.e., the amount of gates being “on”. The redundant parameters can be identified by the zeros (the gates being “off”) in **C**.

Proposition 1 (Proof in Appendix 1). If the projection matrix in Problem 1 is shared between tokens in each field, the optimal solution of Problem 1 can be constructed from one solution to Problem 2.

The solution **d** to Problem 1 can be obtained by setting the size of each token to the number of remaining parameters in its pruned embedding. We note that such constructed **d** satisfies all constraints in Problem 1. First, according to Equation (7), since there are totally at most *k* remaining parameters in the pruned embedding matrix, the constructed **d** meets the budget constraint in Equation (3). Second, the constructed **d** naturally meets the maximal size constraint in Equation (4) since the number of remaining parameters in the pruned embedding are no more than *d*.

As shown in Figure 3, by Proposition 1 and the above analysis, we build the multi-size embedding table for training, where the customized size of each token equals the capacity of its pruned embedding. In the next subsection, we show that Problem 2 can be approximated solved with significant fewer costs.

### 3.2. Prune embeddings without training them

Most of the existing methods in the pruning literature attempt to identify redundant parameters from a pretrained reference network either based on a saliency criterion (Han et al., 2016; Kusupati et al., 2020) or utilizing sparsity enforcing penalties (Carreira-Perpinán and Idelbayev, 2018). Unfortunately, all these pruning methods require many expensive *pretrain-prune-retrain* cycles and introduce additional hyperparameters. Recent work has explored the possibility of pruning neural networks at initialization (Lee et al., 2019; Wang et al., 2020). Namely, given a desired parameter budget, redundant parameters are pruned once before training, and then the pruned network is trained in the standard way. Equipped with the technique, there is no need for network pretraining and complex pruning schedules. Inspired by single-shot network pruning (SNIP) (Lee et al., 2019), we directly prune unimportant parameters in the embedding according to the *connection sensitivity*, which can be obtained by utilizing a full-batch of training data. Consequently, the pruning process is disentangled from the above iterative cycle.

The key idea of *connection sensitivity* proposed in SNIP is to preserve the parameters that have the maximum impact on the loss if perturbed. Specifically, the effect of removing parameter **V**_*i, j*_ on the loss can be measured as follows:


(8) $$\begin{array}{l} \Delta L_{i,j}\left(\text{V},\Theta ;D\right)=L\left(\text{1}⊙\text{V},\Theta ;D\right)-L\left(\left(\text{1}-{\text{e}}_{ij}\right)⊙\text{V},\Theta ;D\right), \end{array}$$

where ${\text{e}}_{ij}\in {\mathbb{R}}^{n\times d}$ is an indicator matrix of element **V**_*i, j*_ (i.e., zeros everywhere except at the *i*^th^ row and *j*^th^ column where it is one), and **1**∈ℝ^*n*×*d*^ is an all-ones matrix. Equation (8) measures the influence of parameter **V**_*i, j*_ on the loss in the discrete setting since **C** is binary. Computing Δ*L*_*i, j*_ for each *i, j* is prohibitively expensive since it requires an individual forward pass over the dataset for each parameter **V**_*i, j*_. However, by relaxing the binary constraint of **C**, Δ*L*_*i, j*_ can be approximated by the derivative of *L* with respect to **C**_*i, j*_, which is named as *connection sensitivity*. Specifically, the *connection sensitivity* **G**(**V**, Θ; D) in SNIP can be computed as follows:


(9) $$\begin{array}{ll} \Delta L_{i,j}\left(\text{V},\Theta ;D\right)\approx {\text{G}}_{i,j}\left(\text{V},\Theta ;D\right) & =\frac{\partial L\left(\text{C}⊙\text{V},\Theta ;D\right)}{\partial {\text{C}}_{i,j}}{|}_{\text{C}=\text{1}} \end{array}$$

(10) $$\begin{array}{l} =\frac{\partial L\left(\text{V},\Theta ;D\right)}{\partial \text{V}}⊙\text{V}. \end{array}$$

Parameters that least impact the performance if removed can be identified according to *connection sensitivity*. We list the full algorithm in Algorithm 1. There is only one hyperparaemter in Algorithm 1, namely, the parameter budget *k*, which controls the total number of parameters in the multi-size table. Specifically, we first initialize a standard single-size embedding layer, then calculate the *connection sensitivity* **G**(**V**, Θ; D). Once **G**(**V**, Θ; D) is obtained, the parameters corresponding to the top-*k* values of |**G**(**V**, Θ; D)| are kept. Finally, the allocated size of each token is set to the number of kept dimensions in its pruned embedding.

**Algorithm 1:**
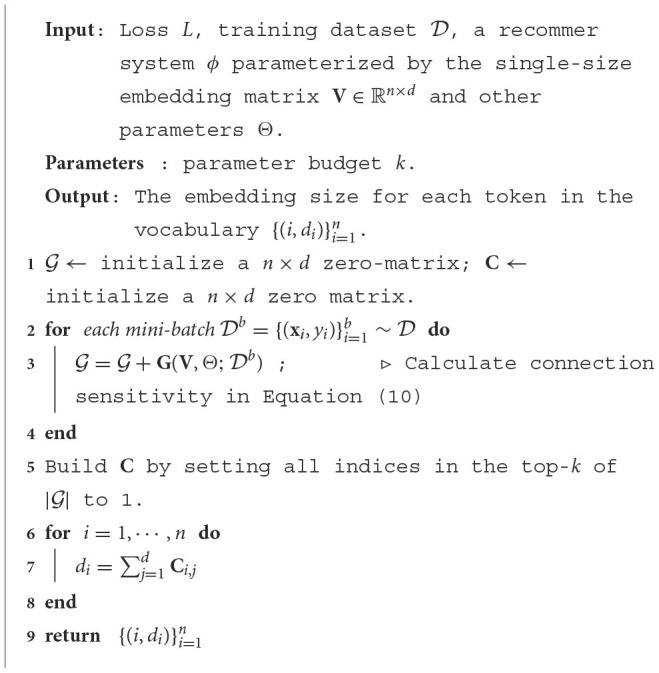
Pruning-base embedding size search.

### 3.3. Multi-size table lookup optimization

Most of the deep learning frameworks do not support embedding table with multiple sizes. In practice, a multi-size table is implemented as multiple two-dimensional matrices, each with different sizes. When retrieving embeddings from a multi-size table, it requires to identify which matrix contains the token's embedding according to its size.

The time cost for identifying the matrix containing the token's embedding grows linearly with the number of candidate matrices. In Algorithm 1, the searched size of each token can be arbitrary integer between 0 and *d*, which means we need to initialize at most *d* two-dimensional matrices. Thus, the retrieval process will be significantly slowed down when *d* is large, which contradicts with the goal of being efficient.

Similar to the previous studies, (Joglekar et al., 2020; Zhao et al., 2020a,b), we define a candidate size set $C=\left\{{\hat{d}}_{1},{\hat{d}}_{2},\cdots \;,{\hat{d}}_{T}\right\}$, where $0\leq {\hat{d}}_{1}<{\hat{d}}_{2}<\cdots <{\hat{d}}_{T}=d$ are *T* predefined embedding sizes. The searched size given by Algorithm 1 will be rounded to its nearest neighbor in C. If ${\hat{d}}_{1}=0$, for these tokens which have been entirely cutoff from the vocabulary (e.g., **v**_3_ in the example of Figure 3), they will be mapped to a padding index. The padding index will then be retrieved as an unlearnable zero vector. Formally, as shown in Figure 2, to retrieve embeddings for a batch of tokens in different fields, we first split them into *T* groups based on their rounded embedding size. Then, we retrieve the embeddings for each group and pad them to equal length with zeros. Finally, we re-arrange these padded embeddings to recover the original order of input tokens, and apply field-specific projection on them. We note that the above padding and retrieving process can be efficiently executed in parallel. As the number of groups *T* is typically small, we found that this group-wise implementation delivers minimal overhead compared with standard single-size embedding.

### 3.4. Discussion and limitation

#### 3.4.1. Discussion

we recap and discuss the difference between our formulation of the embedding size allocation problem and that in a previous study. There are two main difference between them.

First, in most of the previous studies, the size allocation problem is formulated as an architecture selection problem (Joglekar et al., 2020; Zhao et al., 2020a,b). Consequently, following the paradigm of NAS, the validation set is used for selecting the size, i.e., the objective in Equation (1) is $L_{val}\left({\hat{\text{V}}}^{*}\left(\text{d}\right),\Theta^{*}\left(\text{d}\right);D_{val}\right)$. In contrast, we formulate this size allocation problem as a pruning problem, which tries to identify parameters that least impact the training loss if removed. Only with such formulation, we can search embedding sizes without training the model, and hence significantly improve the search efficiency. Moreover, the memory usage of embedding layers can be reduced during both the training and inference phases. A detailed discussion about the difference between the formulation based on NAS and the formulation based on pruning is provided in Appendix 2 (Supplementary material).

Second, most of the previous work constructs several projection matrices for each field. In each field, tokens with same allocated sizes share a common projection matrix. In contrast, we propose to construct only one projection matrix for each field since tokens in a same field have field-level latent structure (Zhao et al., 2020a; Ginart et al., 2021). Specifically, embeddings with different sizes are padded to equal length with zeros, enabling the feasible adoption of the field-specific projections. This approach has nice algebraic explanation (see Equation 5). We note that our approach also enables embeddings of equal length but belonging to different fields to be retrieved simultaneously, which is inflexible in most of the previous studies. A detailed analysis is provided in Appendix 2 (Supplementary material).

#### 3.4.2. Limitation

The main limitation of PME is that, during the embedding size search phase, the memory usage of embedding layers cannot be reduced. However, we note that most of the search based multi-size embedding frameworks also have this problem (Joglekar et al., 2020; Zhao et al., 2020a,b; Liu et al., 2021). It is necessary to initialize embeddings with maximal size to evaluate whether the maximal available size in the search space is suitable for a specific token. In this article, we mainly focused on reducing the memory usage of models during the training and inference phases, and their storage requirements.

## 4. Experiment

We verify the effectiveness of our proposed framework through answering the following research questions:

- **RQ1**. How is PME compared with other embedding compression methods in terms of model performance at different compression rates?
- **RQ2**. What is the additional time cost for searching the embedding size and for training the model, respectively?
- **RQ3**. How sensitive are the searched embedding sizes to the backbone models and to the initialized weights, respectively?

### 4.1. Experimental settings

We first introduce the baseline methods for comparison. Then, we introduce the applied datasets and the hyperparameter settings.

#### 4.1.1. Baselines

We compare our proposed method with the following five representative embedding compression methods: (1) **SE** (single-size embedding): a standard single-size embedding method that assigns a fixed embedding size to all tokens in the vocabulary. (2) **MDE** (mixed dimension embedding) (Ginart et al., 2021): a multi-size embedding method that scales token's embedding sizes with its frequency according to heuristic rules designed by human experts. (3) **QREMB** (quotient-remainder embedding) (Shi et al., 2020): a hashing-based method to reduce the total vocabulary size by storing multiple smaller embedding tables based on a standard remainder-hashing function. (4) **LRF** (low-rank factorization) (Koren et al., 2009): a low-rank based method that factorizes the embedding matrix **V**∈ℝ^*n*×*d*^ as **QR**, where **Q**∈ℝ^*n*×*r*^, **R**∈ℝ^*r*×*d*^, and *r* is the rank, which satisfies *r*<*d*. (5) **DartsEMB** (Zhao et al., 2020b): a NAS-based mutli-size embedding method that relaxes the discrete embedding size allocation problem to a continuous one that can be solved by gradient descent (Liu et al., 2019). This method is chosen to display the performance of NAS-based mutli-size embedding methods.^2^ Different embedding compression methods are deployed to three representative state-of-the-art recommendation models: DeepFM (Guo et al., 2017), Autoint+ (Song et al., 2019) and Wide and Deep (Cheng et al., 2016), to compare their performance. More details about the hyperparameters of these three recommendation models are elaborated in Appendix 3.2 (Supplementary material). Logloss and AUC score are selected as the core metrics for evaluating recommendation model performance.

#### 4.1.2. Data preprocessing

We adopt two public benchmark datasets in this article, i.e., **Criteo**^3^ and **Avazu**.^4^ The basic statistics of these two datasets are summarized in Supplementary Table A1 (Supplementary material). Both the datasets are processed based on the method and codes provided in the study by Song et al. (2019). Following the studies by Guo et al. (2017) and Song et al. (2019), for each dataset, we divide the data into the training (80%), validation (10%), and test sets (10%).

#### 4.1.3. Hyperparameter settings

Since there is a trade-off between recommendation model performance and the number of parameters in the embedding table, to fairly compare the effectiveness of different embedding compression methods, we adjust their hyperparameters to ensure the number of their trainable parameters are comparable. For **PME**, the size of the full SE embedding table to be pruned is set to 32. As illustrated in Section 3, PME has two hyperparameters, namely, the parameter budget *k* and the candidate embedding size set C. The candidate size set C is set to {0, 2, 8, 16, 32} across all experiments, i.e., each searched size given by Algorithm 1 will be rounded to its nearest neighbor in C. Suppose before pruning, the total number of parameters in the single-size embedding table is *K*. The parameter budget *k* is set to 2% × *K*, 5% × *K*, and 10% × *K*. Due to the page limit, detailed hyperparameter settings for all other baselines are specified in Appendix 3.3 (Supplementary material). The **compression rate** *cr* can be calculated as follows:


$$\begin{array}{l} cr=\frac{\text{\# of parameters in the full SE embedding table}}{\text{\# of parameters in the compressed embedding table.}} \end{array}$$

We implement our method using *Pytorch* (Paszke et al., 2019). Every single experiment is run on a single NVIDIA GeForce RTX 1080 Ti GPU with several models parallelly trained on it. To reduce the variance, all of the reported numbers are averaged over four random trials.

### 4.2. Performance vs. parameter number

To answer **RQ1**, we evaluate model performance with embedding compression methods at different compression rates. In addition, we also experimentally analyze the relationship between token's assigned sizes and its frequency to understand how PME allocates embedding sizes for each token.

#### 4.2.1. Criteo and Avazu results

**Figure 4:**
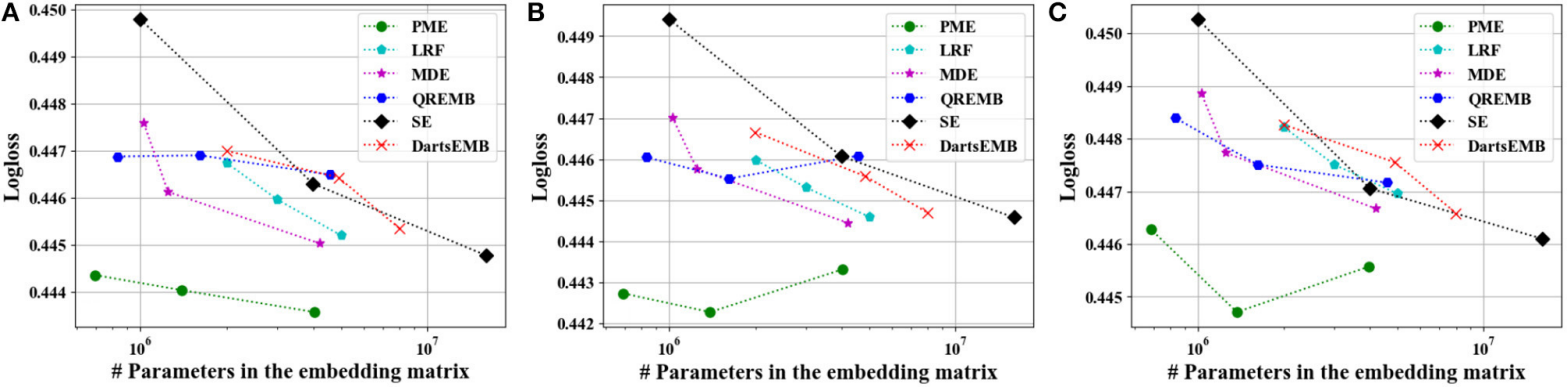
Test Logloss of recommendation models at approximately 10×, 20×, and 40× compression rate on Criteo dataset. **(A)** The backbone model is DeepFM. **(B)** The backbone model is Autoint+. **(C)** The backbone model is Wide and Deep.

**Figure 5:**
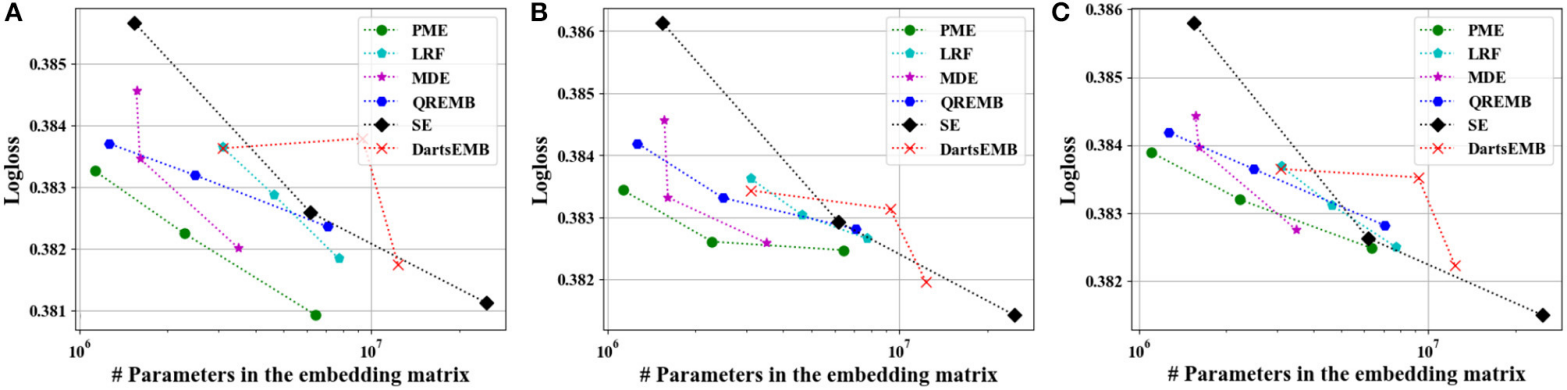
Test Logloss of recommendation models at approximately 10×, 20×, and 40× compression rate on Avazu dataset. **(A)** The backbone model is DeepFM. **(B)** The backbone model is Autoint+. **(C)** The backbone model is Wide and Deep.

**Figure 6:**
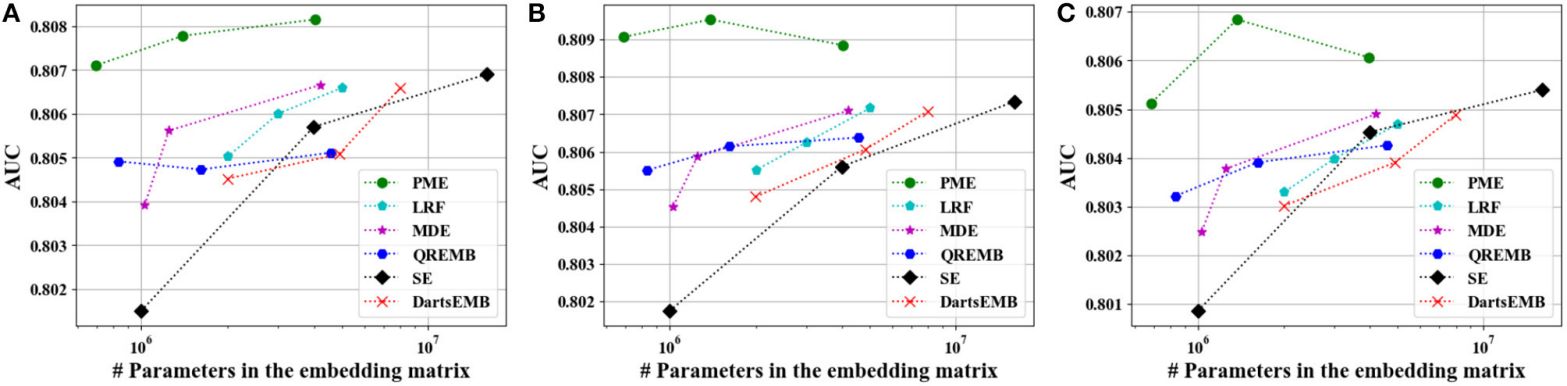
Test AUC of recommendation models at approximately 10×, 20×, and 40× compression rate Criteo dataset. **(A)** The backbone model is DeepFM. **(B)** The backbone model is Autoint+. **(C)** The backbone model is Wide and Deep.

**Figure 7:**
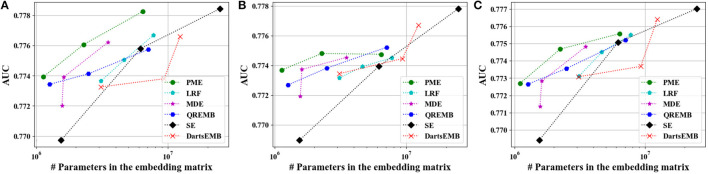
Test AUC of recommendation models at approximately 10×, 20×, and 40× compression rate on Avazu dataset. **(A)** The backbone model is DeepFM. **(B)** The backbone model is Autoint+. **(C)** The backbone model is Wide and Deep.

Figures 4, 5 depict the Logloss of three recommendation models with embedding compression methods on Criteo and Avazu dataset, respectively. We observe that PME generally outperforms other baselines at different compression rates. Furthermore, we remark that PME can outperform SE even when SE uses maximal sizes on Criteo dataset. For example, PME improve the Logloss by 0.001 level while eliminating 97.4% and 95.7% parameters in the embedding layer for Autoint+ and Wide and Deep on Criteo dataset, respectively. It is worth pointing out that an improvement of approximately 0.001 in terms of Logloss or AUC is already regarded as practically significant on these CTR prediction tasks (Cheng et al., 2016). The AUC results are shown in Figures 6, 7, which are similar to the Logloss, due to the page limit. We note that DartsEMB cannot assign zero dimension to tokens due to its NAS-based formulation. Moreover, DartsEMB cannot directly control the compression rate. Consequently, the only way to control the DartsEMB's compression rate is to decrease the maximal available size in its search space. However, decreasing maximal available size will limit the capacity of important tokens' representation. Thus, with DartsEMB, it is hard to achieve good performance at a high compression rate beyond 10 ×. In contrast, PME can directly exclude unimportant tokens from the vocabulary by assigning zero dimensions to them. Since the majority of tokens in the vocabulary are unimportant, PME can maintain the model performance even at an extremely high compression ratio, such as 40 ×. Moreover, we emphasize that the memory usage of recommendation models with PME is reduced during both the standard training and inference process.

#### 4.2.2. Relationship between frequency and allocated sizes

Recent work hypothesizes that frequent tokens are more important for model performance, and hence deserve to have more capacity while few parameters are enough for infrequent tokens (Joglekar et al., 2020; Kang et al., 2020; Ginart et al., 2021). Based on the hyperthesis, several studies explicitly scale the embedding size with token's frequency (Kang et al., 2020; Ginart et al., 2021). In contrast to them, PME learns embedding sizes by transferring the capacity of tokens' pruned embeddings without using the frequency information.

**Figure 8:**
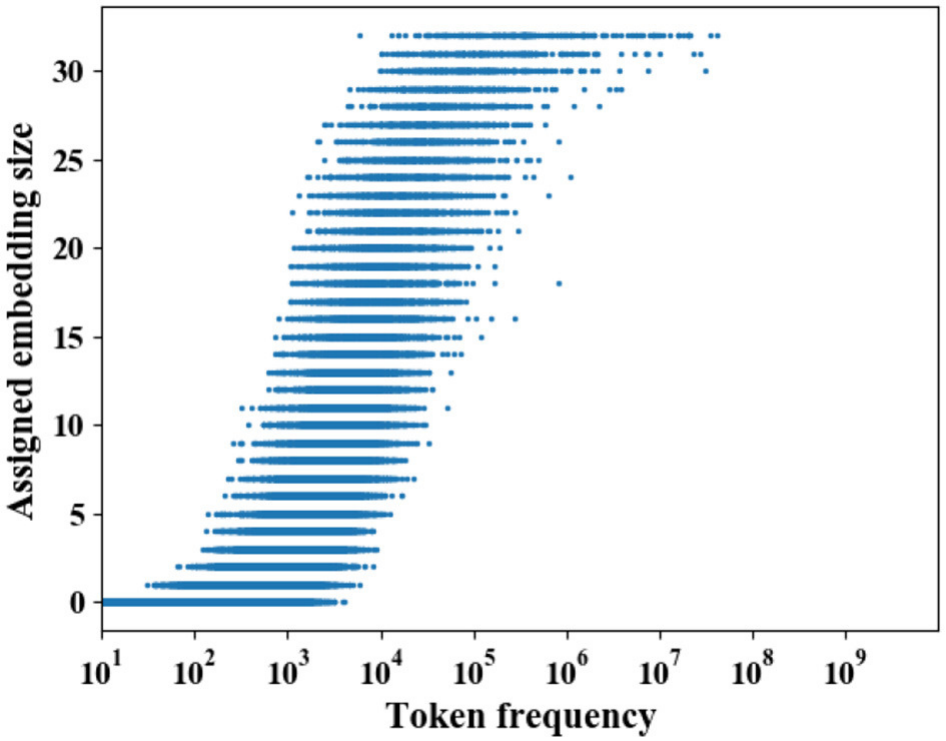
Distribution of token's allocated embedding size across all fields on Criteo Dataset. The backbone model is DeepFM. PME generally assigns larger embedding sizes to frequent tokens and smaller sizes to infrequent tokens.

To study whether the embedding sizes assigned by PME are relevant to the frequency, we visualize the distribution of token's embedding size against its frequency on Criteo dataset in Figure 8, where the backbone model is DeepFM with a 40 × compressed embedding layer. Two main observations are summarized as follows: (1) PME generally assigns larger sizes to frequent tokens, and vice versa. (2) Several infrequent tokens, whose frequency is less than 10^3^, are assigned with large capacity, and some frequent tokens are assigned with a smaller capacity. These two observations are partially aligned with the hyperthesis that frequent tokens are more important for model performance, and hence deserve to have more capacity. More importantly, our observations also suggest that the token's capacity should not be purely decided by its popularity. For example, niche items, such as cult films in movie recommendation, are rarely observed compared with popular ones in the collected data, however, the quality of these niche items' representations is crucial for personalized recommendations, and hence deserve to have more capacity. However, simply scaling embedding sizes with token's frequency may sacrifice the quality of these niche item's representation. In contrast, PME allocates sizes which can maintain model performance with the full embedding as much as possible, and hence may allocate more capacity for tokens whose representation plays a decisive role for recommendation performance.

### 4.3. Efficiency analysis

As shown in Figure 2, the entire pipeline has two phases, namely, the size search phase and the training phase. To answer **RQ2**, we present and analyze the time cost of these two phases, respectively.

For the search phase, we report the search time of PME and DartsEMB in Table 1. We note that all other baselines do not have a separate search process. The search cost of PME is approximately 30%~40% of DartsEMB. This is mainly because the embedding table in PME is not trained during the search. In contrast, DartsEMB follows the paradigm of neural architecture search, leading to solve the bi-level optimization problem during the search.

**Table 1:** Search time (second) of PME and DartsEMB on criteo dataset with different backbone models.

**Search time**	**DeepFM**	**Autoint+**	**Wide and deep**
DartsEMB	801	2,404	745
PME	228 (−71.5%)	1,034 (−60.0%)	219 (−70.6%)

**Figure 9:**
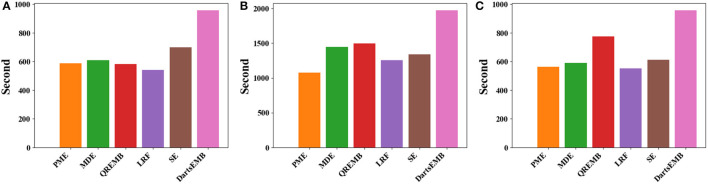
Training time per epoch of recommendation models with different embedding compression methods on Criteo dataset. **(A)** The backbone model is DeepFM. **(B)** The backbone model is Autoint+. **(C)** The backbone model is Wide and Deep.

For the training phase, Figure 9 displays the training time per epoch of three models with different embedding compression methods. We can observe that PME generally reduce the 10%~20% training time compared with SE, and is comparable or faster than other baselines. This speedup may be due to models with PME have significantly less trainable parameters, i.e., many tokens are mapped to unlearnable zero vectors during training (see Figure 8). We remark that PME could retrieve tokens' embeddings from different fields simultaneously, which cannot be done in DartsEMB (see Appendix 2 in Supplementary material). To summarize, PME can not only reduce the memory occupied by the embedding layer during both the training and inference process, but also can speed up the training process.

### 4.4. Sensitivity analysis

In this subsection, we study the sensitivity of searched sizes proposed by PME on backbone models and initialized weights using the Criteo dataset (**RQ3**).

#### 4.4.1. Initialization sensitivity analysis

The Lottery Ticket Hypothesis (LTH) demonstrates randomly initialized networks contain subnetworks (winning tickets) that, when trained in isolation, can reach the accuracy comparable to the original network (Frankle and Carbin, 2019). LTH suggests the connections of winning tickets have those specific initial weights that make training particularly effective (Frankle and Carbin, 2019).

**Figure 10:**
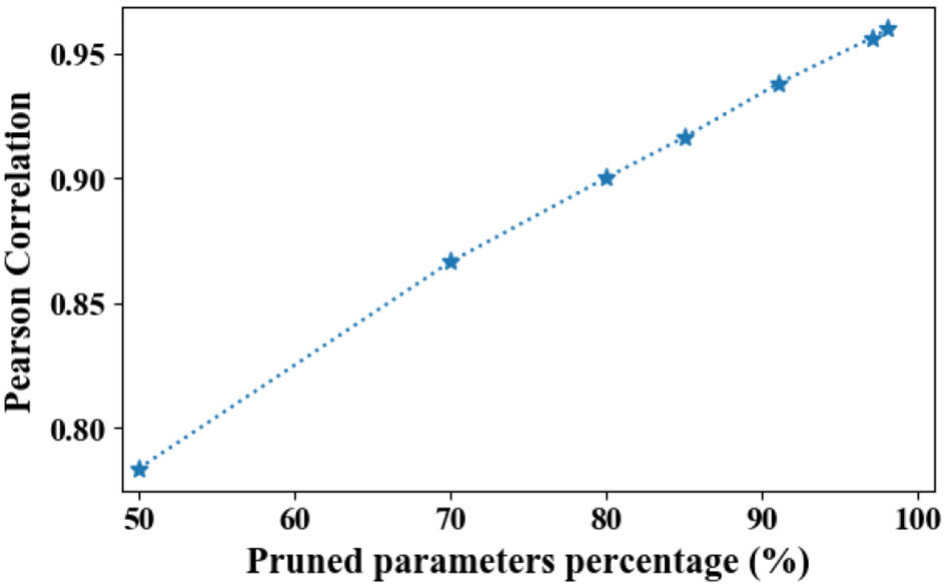
Averaged Pearson correlation between searched sizes with different random seeds. As parameters are being pruned, the Pearson correlation converges to one.

However, in PME, the allocated size of each token is obtained by transferring only the capacity of its pruned embedding. Moreover, the randomly initialized weights used for identifying redundant parameters are not trained during the search process. According to LTH, the allocated sizes may overfit the particular initialized weights used during the search process. To investigate whether searched sizes are customized for the initialized weights used during the search process, following the method given in the study by Zhao et al. (2020a), we calculate the averaged Pearson correlation of searched sizes with five different random seeds. Here, the searched sizes refers to the output of Algorithm 1, instead of rounded sizes for a fine-grained comparison. The results are presented in Figure 10. We note that a Pearson correlation beyond 0.8 is already regarded as strongly correlated (Buda and Jarynowski, 2010; Zhao et al., 2020a).

As shown in Figure 10, PME is generally robust to different initializations in terms of Pearson correlation. Moreover, as the parameters are being pruned, the Pearson correlation converges to one. This suggests that under highly limited resource constraints, the allocation strategy of PME is initialization-agnostic.

#### 4.4.2. Architecture sensitivity analysis

**Figure 11:**
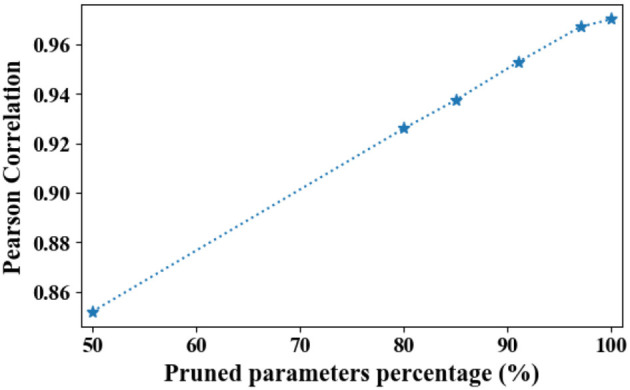
Averaged Pearson correlation between searched sizes with DeepFM and Autoint+. Here, we use the searched sizes instead of rounded sizes. As parameters are being pruned, the Pearson correlation converges to one.

For PME, the embedding sizes are calculated based on the gradients of the randomly initialized weights. Thus, backbone models may largely influence the searched embedding sizes since the gradient flow is decided by the architecture of backbone model. To investigate whether the searched embedding sizes are sensitive to the backbone models, similar to the initialization sensitivity analysis experiments, Figure 11 presents the Pearson correlation of searched embedding sizes with two representative models, namely, DeepFM and Autoint+.

Similarly, as shown in Figure 11, PME is generally robust to backbone models in terms of Pearson correlation. Moreover, as the parameters are being pruned, the Pearson correlation converges to one. This suggests that under highly limited resource constraints, the searched embedding sizes proposed by PME is model-agnostic. We note that both DeepFM and Autoint+ with PME can achieve comparable or better performance at high compression rates on Criteo dataset (see Figure 4), we hypothesize that although backbone models are different, PME identifies a same group of the most important tokens and allocate more parameters to them.

## 5. Related work

Many embedding compression embedding methods have been proposed to reduce the memory consumption of the embedding layer. We roughly categorize existing embedding compression methods into four classes as follows.

### 5.1. Multi-size embedding

Multi-size embedding allows each token in the vocabulary to have embeddings of different sizes. Specifically, mixed dimension embedding (MDE) proposes to adaptively allocate sizes for tokens according to their frequency (Ginart et al., 2021). Neural Input Search (NIS) tries to search the embedding size using Reinforcement Learning (Joglekar et al., 2020). Inspired by the differentiable architecture search (DARTS) (Liu et al., 2019), AutoEmb makes the embedding sizes selection process differentiable by incorporating the DARTS method (Zhao et al., 2020b). Similarly, AutoDim proposes to search field-wise embedding sizes by relaxing the discrete embedding size allocation problem to a continuous one that can be solved by gradient descent (Zhao et al., 2020a).

Plug-in Embedding Pruning (PEP) (Liu et al., 2021) also adopts the pruning-based formulation to learn embedding sizes, which is the most related study to ours with two main differences. First, PEP uses the sparse matrix format to store the pruned embedding layer and retrains the model with the sparse embedding matrix. In contrast, PME builds a multi-size embedding table for training by transferring the capacity of the token's pruned embeddings. Second, PEP utilizes Soft Threshold Reparameterization (Kusupati et al., 2020) to prune redundant parameters, which requires expensive *pretrain*-*prune*-*retrain* cycles. In contrast, PME disentangles the pruning process from the iterative cycle by pruning redundant parameters at initialization. We do not compare with PEP due to the following two reasons. First, to the best of our knowledge, the official implementation of embedding layers in Pytorch does not support the sparse matrix format. The official codes of PEP have not released yet. Second, the baseline performance reported in Liu et al. (2021) has a large gap with ours.

### 5.2. Low-rank approximation

Low-rank approximation assumes there is a low-rank latent structure in the embedding matrix, and decomposes the original matrix to several smaller matrices (Markovsky and Usevich, 2012). TT-Rec uses tensor train decomposition instead of the standard low-rank decomposition to optimize for GPU computations (Yin et al., 2021).

### 5.3. Hashing

Hashing is a widely used technique to reduce the store space by mapping similar tokens into the same bucket, and vice versa (Wang et al., 2017). Recently, efforts have also been devoted to jointly learn feature representations and hashing functions to preserve the similarity, and hence minimize the performance gap after compression (Lin et al., 2015; Cao et al., 2017; Wang et al., 2017). Another representative work is ROBE (Desai et al., 2022). Specifically, Desai et al. (2022) maintain a single array for learned parameters which is a compressed representation of embedding table. All embedding tables share the same array of learned parameters. The embeddings are accessed in a blocked manner from the embedding array using GPU-friendly universal hashing.

### 5.4. Quantization

Quantization refers to representing weights or gradients with a small numbers of bits, e.g., eight bits. In this way, we can effectively shrink the model size and accelerate the inference procedures (Han et al., 2016). Specifically, differentiable product quantization (DPQ) proposes a differentiable quantization framework that enables end-to-end training for embedding compression and achieves significant compression rates on NLP models (Chen et al., 2020). Inspired by DPQ, multi-granular quantized embeddings (MGQEs) generalize the framework of DPQ to the recommendation domain by incorporating the frequency information of tokens (Kang et al., 2020).

## 6. Conclusion

In this study, we approach the embedding size allocation problem from a pruning perspective. During the search phase, we prune the dimensions that have the least impact on model performance in the embedding to reduce its capacity. Then, we show that the customized size of each token can be obtained by transferring the capacity of its pruned embedding. Experiments verify that PME can achieve strong performance while significantly reducing the parameter number and can be trained efficiently.

## Data availability statement

The original contributions presented in the study are included in the article/Supplementary material, further inquiries can be directed to the corresponding author.

## Author contributions

ZL, QS, and XH contributed to the whole framework. XH, QS, LL, S-HC, and RC contributed to the revision of the manuscript. All authors contributed to the manuscript and approved the submitted version.

## Footnotes

^1^For convenience, we use the term “tokens” to represent elements (e.g., users and items) in the vocabulary.

^2^We do not compare with NIS (Joglekar et al., 2020), since the reinforcement learning based search process is extremely slow in the normal setting.

^3^ https://www.kaggle.com/c/criteo-display-ad-challenge

^4^ https://www.kaggle.com/c/avazu-ctr-prediction

## References

[B1] Buda A. Jarynowski A. (2010). Life Time of Correlations and its Applications. Andrzej Buda Wydawnictwo NiezaleĹL'ne.

[B2] Cao Z. Long M. Wang J. Yu P. S. (2017). “Hashnet: Deep learning to hash by continuation,” in IEEE International Conference on Computer Vision, ICCV 2017 (Venice: IEEE Computer Society), 5609–5618. 10.1109/ICCV.2017.598

[B3] Carreira-Perpinán M. A. Idelbayev Y. (2018). ““Learning-compression” algorithms for neural net pruning,” in Proceedings of the IEEE Conference on Computer Vision and Pattern Recognition, 8532–8541.

[B4] Chen T. Li L. Sun Y. (2020). “Differentiable product quantization for end-to-end embedding compression,” in Proceedings of the 37th International Conference on Machine Learning, ICML 2020, Virtual Event (PMLR), vol. 119 of Proceedings of Machine Learning Research (Vienna), 1617–1626.

[B5] Cheng H.-T. Koc L. Harmsen J. Shaked T. Chandra T. Aradhye H.. (2016). “Wide & deep learning for recommender systems,” in Proceedings of the 1st Workshop on Deep Learning for Recommender Systems, 7–10.

[B6] Covington P. Adams J. Sargin E. (2016). “Deep neural networks for youtube recommendations,” in Proceedings of the 10th ACM Conference on Recommender Systems, eds Sen S. Geyer W. Freyne J. Castells P. (Boston, MA: ACM), 191–198. 10.1145/2959100.2959190

[B7] Desai A. Chou L. Shrivastava A. (2022). “Random offset block embedding (ROBE) for compressed embedding tables in deep learning recommendation systems,” in Proceedings of Machine Learning and Systems 2022, MLSys 2022, eds Marculescu D. Chi Y. Wu C. (Santa Clara, CA: mlsys.org).

[B8] Frankle J. Carbin M. (2019). “The lottery ticket hypothesis: Finding sparse, trainable neural networks,” in 7th International Conference on Learning Representations, ICLR 2019 (New Orleans, LA: OpenReview.net).

[B9] Ginart A. A. Naumov M. Mudigere D. Yang J. Zou J. (2021). “Mixed dimension embeddings with application to memory-efficient recommendation systems,” in IEEE International Symposium on Information Theory, ISIT 2021 (Melbourne, VIC: IEEE), 2786–2791. 10.1109/ISIT45174.2021.9517710

[B10] Guo H. Tang R. Ye Y. Li Z. He X. (2017). “Deepfm: A factorization-machine based neural network for CTR prediction,” in Proceedings of the Twenty-Sixth International Joint Conference on Artificial Intelligence, IJCAI 2017, ed Sierra C. (Melbourne, VIC: ijcai.org), 1725–1731. 10.24963/ijcai.2017/239

[B11] Han S. Mao H. Dally W. J. (2016). “Deep compression: Compressing deep neural network with pruning, trained quantization and huffman coding,” in 4th International Conference on Learning Representations, ICLR 2016, eds Y. Bengio and Y. LeCun (San Juan).

[B12] Joglekar M. R. Li C. Chen M. Xu T. Wang X. Adams J. K.. (2020). “Neural input search for large scale recommendation models,” in KDD '20: The 26th ACM SIGKDD Conference on Knowledge Discovery and Data Mining, Virtual Event, eds R. Gupta, Y. Liu, J. Tang, and B. A. Prakash (New York, NY: ACM), 2387–2397. 10.1145/3394486.3403288

[B13] Kang W. Cheng D. Z. Chen T. Yi X. Lin D. Hong L.. (2020). “Learning multi-granular quantized embeddings for large-vocab categorical features in recommender systems,” in Companion of The 2020 Web Conference 2020, eds A. E. F. Seghrouchni, G. Sukthankar, T. Liu, and M. van Steen (Taipei: ACM / IW3C2), 562–566. 10.1145/3366424.3383416

[B14] Koren Y. Bell R. Volinsky C. (2009). Matrix factorization techniques for recommender systems. Computer 42, 30–37. 10.1109/MC.2009.263

[B15] Kusupati A. Ramanujan V. Somani R. Wortsman M. Jain P. Kakade S. M.. (2020). “Soft threshold weight reparameterization for learnable sparsity,” in Proceedings of the 37th International Conference on Machine Learning, ICML 2020, Virtual Event (PMLR), vol. 119 of Proceedings of Machine Learning Research, 5544–5555.

[B16] Lee N. Ajanthan T. Torr P. H. S. (2019). “Snip: single-shot network pruning based on connection sensitivity,” in 7th International Conference on Learning Representations, ICLR 2019 (New Orleans, LA: OpenReview.net).

[B17] Lian J. Zhou X. Zhang F. Chen Z. Xie X. Sun G. (2018). “xdeepfm: Combining explicit and implicit feature interactions for recommender systems,” in Proceedings of the 24th ACM SIGKDD International Conference on Knowledge Discovery & Data Mining, KDD 2018, eds Y. Guo and F. Farooq (London: ACM), 1754–1763. 10.1145/3219819.3220023

[B18] Lin K. Yang H. Hsiao J. Chen C. (2015). “Deep learning of binary hash codes for fast image retrieval,” in 2015 IEEE Conference on Computer Vision and Pattern Recognition Workshops, CVPR Workshops 2015 (Boston, MA: IEEE Computer Society), 27–35. 10.1109/534CVPRW.2015.7301269

[B19] Liu H. Simonyan K. Yang Y. (2019). “DARTS: differentiable architecture search,” in 7th International Conference on Learning Representations, ICLR 2019 (New Orleans, LA: OpenReview.net).

[B20] Liu S. Gao C. Chen Y. Jin D. Li Y. (2021). “Learnable embedding sizes for recommender systems,” in 9th International Conference on Learning Representations, ICLR 2021, Virtual Event (OpenReview.net).

[B21] Markovsky I. (2012). “Low rank approximation - algorithms, implementation, applications,” in Communications and Control Engineering (London: Springer). 10.1007/978-1-4471-2227-2

[B22] Park J. Naumov M. Basu P. Deng S. Kalaiah A. Khudia D. S.. (2018). Deep learning inference in facebook data centers: Characterization, performance optimizations and hardware implications. arXiv [Preprint]. arXiv: 1811.09886.

[B23] Park Y. Tuzhilin A. (2008). “The long tail of recommender systems and how to leverage it,” in Proceedings of the 2008 ACM Conference on Recommender Systems, RecSys 2008, eds P. Pu, D. G. Bridge, B. Mobasher, and F. Ricci (Lausanne: ACM), 11–18. 10.1145/1454008.1454012

[B24] Paszke A. Gross S. Massa F. Lerer A. Bradbury J. Chanan G.. (2019). “Pytorch: An imperative style, high-performance deep learning library,” in Advances in Neural Information Processing Systems 32: Annual Conference on Neural Information Processing Systems 2019, NeurIPS 2019, eds H. M. Wallach, H. Larochelle, A. Beygelzimer, F. d'Alché–Buc, E. B. Fox, and R. Garnett (Vancouver, BC), 8024–8035.

[B25] Shi H. M. Mudigere D. Naumov M. Yang J. (2020). “Compositional embeddings using complementary partitions for memory-efficient recommendation systems,” in KDD '20: The 26th ACM SIGKDD Conference on Knowledge Discovery and Data Mining, Virtual Event, eds Gupta R. Liu Y. Tang J. Prakash B. A. (ACM), 165–175. 10.1145/3394486.3403059

[B26] Song W. Shi C. Xiao Z. Duan Z. Xu Y. Zhang M.. (2019). “Autoint: Automatic feature interaction learning via self-attentive neural networks,” in Proceedings of the 28th ACM International Conference on Information and Knowledge Management, CIKM 2019, eds W. Zhu, D. Tao, X. Cheng, P. Cui, E. A. Rundensteiner, D. Carmel, Q. He, and J. X. Yu (Beijing: ACM), 1161–1170. 10.1145/3357384.3357925

[B27] Wang C. Zhang G. Grosse R. B. (2020). “Picking winning tickets before training by preserving gradient flow,” in 8th International Conference on Learning Representations, ICLR 2020 (Addis Ababa: OpenReview.net).

[B28] Wang J. Zhang T. Sebe N. Shen H. T. (2017). A survey on learning to Hash. IEEE Trans. Pattern Anal. Mach. Intell. 40, 769–790. 10.1109/TPAMI.2017.2699960 28475044

[B29] Yin C. Acun B. Wu C. Liu X. (2021). “Tt-rec: Tensor train compression for deep learning recommendation models,” in Proceedings of Machine Learning and Systems 2021, MLSys 2021, virtual, eds Smola A. Dimakis A. Stoica I. (mlsys.org).

[B30] Zhang S. Yao L. Sun A. Tay Y. (2019). Deep learning based recommender system: a survey and new perspectives. ACM Comput. Surv. 52, 1–38. 10.1145/3158369

[B31] Zhao X. Liu H. Liu H. Tang J. Guo W. Shi J.. (2020a). Memory-efficient embedding for recommendations. arXiv [Preprint]. arXiv:2006.14827.

[B32] Zhao X. Wang C. Chen M. Zheng X. Liu X. Tang J. (2020b). AutoEMB: automated embedding dimensionality search in streaming recommendations. arXiv preprint arXiv:2002.11252.

