# Supplementary material for: PME: pruning-based multi-size embedding for recommender systems
Source: Front Big Data. 2023 Jun 15;6:1195742. doi: 10.3389/fdata.2023.1195742 (PMC10311001; doi:10.3389/fdata.2023.1195742)
Supplement: Supplementary file 1 [file Data_Sheet_1.pdf]

frontiers

Supplementary Material

1 PROOF OF PROPOSITION 1

PROPOSITION 1 (Proof in Appendix 1). *If the projection matrix in Problem 1 is shared between tokens in each field, the optimal solution of Problem 1 can be constructed from one solution to Problem 2.*

PROOF. We prove this statement by construction. We first prove it for the case that there is only one field. Since the projection matrix is shared between tokens in the field, from the definition of $\hat{\mathbf{V}}$, we have $\forall i, \hat{\mathbf{V}}_{i,:} = \mathbf{P}\hat{\mathbf{v}}'_i$. We note that this is equivalent to $\hat{\mathbf{V}} = \hat{\mathbf{V}}'\mathbf{P}$, where $\hat{\mathbf{V}}' \in \mathbb{R}^{n\times d}$ is the sparse matrix obtained by stacking the padded embedding vectors $\hat{\mathbf{v}}'_i$, i.e., $\forall i, \hat{\mathbf{V}}'_{i,:} = \hat{\mathbf{v}}'_i$.

Suppose an optimal solution to Problem 1 is $\mathbf{d}^*$, $\hat{\mathbf{V}}^*$, Θ^*, and the projection matrix to this field is $\mathbf{P}^*$, where $\hat{\mathbf{V}}^* = \hat{\mathbf{V}}'^*\mathbf{P}^*$. To prove $\mathbf{d}^*$, $\hat{\mathbf{V}}^*$, Θ^* lies in the search space of Problem 2, we need to show there exists a $\mathbf{C}$, $\mathbf{V}$, and Θ, such that (i) $\mathcal{L}(\hat{\mathbf{V}}^*, \Theta^*; \mathcal{D}) = \mathcal{L}(\mathbf{V} \odot \mathbf{C}, \Theta; \mathcal{D})$; (ii) $\mathbf{C}$ satisfies the sparse constraint in Equation (7).

We can construct a specific $\mathbf{V}$ and $\mathbf{C}$ in Equation (6) to satisfy $\mathbf{V} \odot \mathbf{C} = \hat{\mathbf{V}}'^*$. The gate in the constructed $\mathbf{C}$ is set as "off" if its corresponding parameter in $\hat{\mathbf{V}}'^*$ is a padding zero, and set as "on" for other parameters. For the constructed $\mathbf{C}$, we have:

$$||\mathbf{C}||_0 = \sum \mathbf{d}^* = \sum_{i=1}^{n} d_i^* \tag{S1}$$

Since $\mathbf{d}^*$ is a solution to Problem 1, we have $\sum_{i=1}^{n} d_i^* \leq k$. Thus, the constructed $\mathbf{C}$ meets the sparse constraint in Equation (7). Note that $\mathbf{V} \odot \mathbf{C} = \hat{\mathbf{V}}'^* \neq \hat{\mathbf{V}}^*$, we will show $\mathcal{L}(\hat{\mathbf{V}}^*, \Theta^*; \mathcal{D}) = \mathcal{L}(\mathbf{V} \odot \mathbf{C}, \Theta; \mathcal{D})$ by constructing a specific Θ in the next step.

We have $\hat{\mathbf{V}}^* = \hat{\mathbf{V}}'^*\mathbf{P}^*$ and $\mathbf{V} \odot \mathbf{C} = \hat{\mathbf{V}}'^*$. In problem 1, the projection operation specified by $\mathbf{P}^*$ is implemented as adding a linear layer to the transform the retrieved embeddings of this field, where the weight of this linear layer equals $\mathbf{P}^*$. Similarly, for the model in Problem 2, we can construct the corresponding linear layer for this field by setting its weights as $\mathbf{P}^*$. Consequently, by setting $\Theta = \mathbf{P}^* \cup \Theta^*$, we have $\mathcal{L}(\hat{\mathbf{V}}^*, \Theta^*; \mathcal{D}) = \mathcal{L}(\mathbf{V} \odot \mathbf{C}, \Theta; \mathcal{D})$. Thus, we prove the statement for the case where there is only one field. If there are multiple fields, suppose the projection matrix for m^{th} field is $\mathbf{P}_m^*$, then we can obtain the same result by constructing a linear layer for each field, where the weight matrix is set as $\mathbf{P}_m^*$.

2 DETAILS FOR SECTION 3.4

2.1 NAS-based Formulation and Pruning-based Formulation

Most of the previous multi-size embedding work formulates the size allocation problem as an architecture selection problem. Consequently, following the paradigm of NAS, the validation data is used to select the size. The main reason to use validation data is to prevent the trivial solution. Namely, if the training data is used to select the size, all tokens may be allocated with maximal available sizes since using all parameters may have the lowest training loss. However, it has been reported in previous paper that this statement may

not true for size allocation problem Liu et al. (2021). In Liu et al. (2021), the authors found that the training loss can be further minimized if some unimportant tokens are allocated with less capacity.

In contrast, we formulate this size allocation problem as a pruning problem. That is to identify parameters least degrade model performance on the training data if removed. In our framework, the customized embedding sizes are searched merely on the training data. However, our multi-size embedding table with customized sizes generalizes well and can even outperform the performance with full embedding table, which is experimentally verified.

Also, NAS based multi-size embedding work has another practical drawback when applying it. Specifically, NAS based formulation requires the data to be divided into the training set and validation set during the search process. However, in recommendation domain, the distribution of tokens is extremely sparse. Some tokens might only appear in the validation set while absent in the training set, and vice versa. Consequently, if a token does not present in the validation set, then NAS based methods may allocate a smallest available size to it, since the token has no relationship with the validation loss. Thus, the splitting may largely influence the searched sizes of NAS based methods.

2.2 Details about Retrieving Embeddings

In most of the previous multi-size embedding work, there are several projection matrices constructed for each field, where tokens with same allocated sizes share a common projection matrix in each field. For embeddings of equal length but belonging to different fields, retrieving them simultaneously is inflexible due to the following reason. To recover the original order of input tokens in a mini-batch, the reorder operation requires the retrieved embeddings to be in equal length for storing in fixed-size containers, such as Tensor or Matrix. However, each projection operation corresponds to a particular size in a specific field, and hence cannot be directly applied to the retrieved embeddings. One flexible solution in previous work is to retrieve embeddings from different field separately. Then apply size-specific projection for retrieved embeddings in each field. However, this approach will significantly slow down the retrieval process.

In contrast, PME constructs only one projection matrix for each field. In particular,embeddings with different sizes are padded to equal length with zeros, enabling the feasible adoption of the field-specific projections. The above padding and retrieving process can be efficiently executed in parallel.

3 DETAILED EXPERIMENTAL SETTINGS

3.1 The Details of Two Benchmark Datasets

In this section, we will introduce the two applied datasets. Table S1 summaries the basic statistics of two datasets.

Table S1. Statistics of Datasets

Dataset	# Samples	# Numerical Feature Fields	# Categorical Feature Fields	Total Cardinality
Criteo	45,840,617	13	26	998,960
Avazu	40,428,967	0	23	1,544,488

- Criteo is a benchmark dataset for evaluating click-through rate prediction tasks (CTR). It contains 45 million users' click records on displayed advertisements.

- Avazu is a benchmark dataset for the CTR prediction task. This dataset contains users' mobile behaviors including whether a displayed mobile advertisement is clicked by a user or not.

3.2 Details about Recommendation Models

The details of the architectures and training hyperparameters of three backbone recommendation models are listed as following:

- DeepFM Guo et al. (2017) : a two-tower model composed of a three-layer multi-layer perceptrons (MLP) with 400 hidden units in each layer and factorization machine block, the embedding layers are shared between the two components, and the output of the two components are linearly embedded with sigmoid transformation as the final prediction
- Autoint+ Song et al. (2019) : a two-tower model composed of a three-layer MLP with 400 hidden units in each layer and three-layer self-interaction layer, which adopts the self-attention schema to learn feature interactions. Within each interaction layer, we follow the default configurations reported in Song et al. (2019), except for setting the number of hidden units to 64 instead of 32. The outputs of the two components are linearly embedded with sigmoid transformation into the final prediction.
- Wide $\&$ Deep Pan et al. (2020) : a two-tower model conposed of a two-layer MLP (the deep part), which has the architecture of 256-128 first-to-last hidden layer sizes, and a linear model (the wide part). The outputs of the two components are linearly embedded with sigmoid transformation into the final prediction. We do not follow the official implementation where the two parts are optimized by different optimizers. For convenience, we use a shared ADAM optimizer Kingma and Ba (2015) for both the deep and wide part.

For all three models, they are trained with learning rate of $1e-3$, batch size of 4096, dropout rate of 0.5, and the Adam optimizer. For the MLP in these three models, ReLU is used as activation functions and the Batch Normalization is adopted. We do not add any other regularizations excluding Wide $\&$ Deep. For Wide $\&$ Deep, a $1e-5$ L_2 regularization is applied.

3.3 Baselines Hyperparameter Settings

For **SE**, we set the fixed embedding size as 1, 8, and 32. For **MDE**, the baseline dimension is set to 32. The temperature α is set to 0.2, 0.3 and 0.4. For **QREMB**, as suggested in Shi et al. (2020), we use the multiplication operation to combine compositional embeddings. The hash collisions is set to 7, 20, and 40. For **LRF**, the rank r is set to 2, 3, and 5. Since **DartsEMB** cannot explicitly control the total number of parameters in the multi-size embedding table, for DartsEMB, the candidate embedding space is set to $\{1,2,8\}$, $\{2,4,16\}$, and $\{4,8,32\}$ to meet different compression rates.

REFERENCES

Guo, H., Tang, R., Ye, Y., Li, Z., and He, X. (2017). Deepfm: A factorization-machine based neural network for CTR prediction. In *Proceedings of the Twenty-Sixth International Joint Conference on Artificial Intelligence, IJCAI 2017, Melbourne, Australia, August 19-25, 2017*, ed. C. Sierra (ijcai.org), 1725–1731. doi:10.24963/ijcai.2017/239

Kingma, D. P. and Ba, J. (2015). Adam: A method for stochastic optimization. In *3rd International Conference on Learning Representations, ICLR 2015, San Diego, CA, USA, May 7-9, 2015, Conference Track Proceedings*, eds. Y. Bengio and Y. LeCun

Liu, S., Gao, C., Chen, Y., Jin, D., and Li, Y. (2021). Learnable embedding sizes for recommender systems. In *9th International Conference on Learning Representations, ICLR 2021, Virtual Event, Austria, May 3-7, 2021* (OpenReview.net)

Pan, Y., He, F., and Yu, H. (2020). Learning social representations with deep autoencoder for recommender system. *World Wide Web* 23, 2259–2279. doi:10.1007/s11280-020-00793-z

Shi, H. M., Mudigere, D., Naumov, M., and Yang, J. (2020). Compositional embeddings using complementary partitions for memory-efficient recommendation systems. In *KDD '20: The 26th ACM SIGKDD Conference on Knowledge Discovery and Data Mining, Virtual Event, CA, USA, August 23-27, 2020*, eds. R. Gupta, Y. Liu, J. Tang, and B. A. Prakash (ACM), 165–175. doi:10.1145/3394486.3403059

Song, W., Shi, C., Xiao, Z., Duan, Z., Xu, Y., Zhang, M., et al. (2019). Autoint: Automatic feature interaction learning via self-attentive neural networks. In *Proceedings of the 28th ACM International Conference on Information and Knowledge Management, CIKM 2019, Beijing, China, November 3-7, 2019*, eds. W. Zhu, D. Tao, X. Cheng, P. Cui, E. A. Rundensteiner, D. Carmel, Q. He, and J. X. Yu (ACM), 1161–1170. doi:10.1145/3357384.3357925
